# Mental Preparation in Professional Tennis: A Qualitative Study of Czech Players’ Experiences

**DOI:** 10.3390/sports14100429

**Published:** 2026-10-03

**Authors:** Vendula Redlichová, Jana Novotná, Martin Komarc

**Affiliations:** Faculty of Physical Education and Sport, Charles University, José Martího 269/31, 162 52 Prague, Czech Republic; jana.novotna@ftvs.cuni.cz (J.N.); martin.komarc@ftvs.cuni.cz (M.K.)

**Keywords:** psychological preparation, professional tennis, coping, emotion regulation, psychological skills training, reflexive thematic analysis

## Abstract

Psychological preparation is increasingly recognized as an important component of athletic performance; however, comparatively little is known about how professional tennis players experience psychological preparation throughout their careers. This qualitative study explored the experiences and subjective interpretations of psychological preparation among Czech professional tennis players. Eight players aged 17–26 years and ranked between 145 and 719 in the ATP/WTA singles rankings were purposively recruited. Semi-structured interviews were conducted in Czech and analyzed using reflexive thematic analysis informed by an experiential and phenomenological orientation. Three themes and seven subthemes were generated: (1) Psychological Preparation Is Valued but Rarely Fully Embraced; (2) Finding and Regaining Psychological Control During Competition; and (3) Managing the Psychological Demands of Professional Tennis. Participants described psychological preparation as important but frequently informal, individually driven, and inconsistently integrated into their development. Personal readiness, confidence, perceived momentum, emotion regulation, pre-match routines, and reflection on competitive outcomes were important aspects of their experiences. Participants also described substantial psychological strain, while social support, broader life perspectives, and personally developed coping strategies supported adaptation to professional tennis. The findings suggest potential value in providing individualized, context-sensitive psychological support and more systematically integrating psychological preparation within professional tennis environments.

## 1. Introduction

As an individual sport, tennis places substantial psychological demands on athletes [1]. Previous research suggests that mental toughness is associated with better performance in several match situations, particularly during critical points requiring effective coping with pressure and adversity [2]. Players are required to make rapid tactical decisions and adapt dynamically, all while balancing intense physical exertion with technical execution and strategic thinking. These cognitive demands can lead to mental fatigue, thereby increasing the likelihood of errors [3]. Furthermore, emotional experiences and their regulation represents important aspects of competitive sport [4]. According to Self-Determination Theory, the satisfaction of basic psychological needs, including relatedness, is important for autonomous motivation and well-being. In the context of professional tennis, maintaining motivation may therefore represent an important psychological consideration [5].

Recent research highlights the importance of psychological factors in sport, with arousal-regulation strategies potentially enhancing performance and athletes’ psychological states influencing subsequent competitive outcomes [6,7]. Meta-analytic evidence further indicates an overall positive association between self-confidence and sport performance, although substantial variability across studies suggests that this relationship is complex and may depend on contextual and individual factors [8]. As a result, integrating psychological skills training alongside physical, technical, and tactical preparation appears relevant in tennis, with evidence from racket sports suggesting beneficial effects of mental training interventions on both performance and psychological outcomes [9,10,11]. Taken together, these psychological demands have a profound impact on performance outcomes and athlete well-being, underscoring the critical role of effective emotion regulation, motivational control, and sustained attentional focus in professional competition.

In recent years, psychological preparation has become increasingly recognized as an essential component of high-level performance in tennis [1,9,12]. Within the context of this study, psychological preparation is understood broadly as the systematic development and application of psychological skills and strategies aimed at helping athletes manage the cognitive and emotional demands of training and competition [10,13]. Despite this growing recognition, psychological support is not always systematically integrated into the wider performance environment and often remains fragmented across stakeholders and services [14].

Importantly, psychological preparation does not occur in isolation but is embedded within athletes’ broader psychological and social environment. While performance-focused psychological skills training is conceptually distinct from clinical mental health support, both contribute to athletes’ capacity to cope with the demands of elite sport. Research indicates that elite athletes are exposed to a range of sport-specific stressors, including performance pressure, injury, relocation, intensive training demands, and major career transitions, all of which may place considerable strain on psychological wellbeing. Consequently, elite athletes frequently report symptoms of psychological distress, including anxiety, depression, sleep disturbances, and other mental health difficulties [15,16]. Contemporary approaches to mental health in elite sport increasingly emphasize the role of psychologically safe environments, in which athletes feel able to disclose difficulties, seek support, and engage with available mental health resources without fear of negative consequences. Psychologically safe and mentally healthy environments have been associated with lower psychological distress and greater well-being among athletes and support staff [17]. Broader evidence-informed frameworks further identify psychological safety, resilience-building, social support, and access to mental health professionals as core components of effective mental health promotion in elite sport [18].

Qualitative research in professional tennis has provided valuable insights into the psychological and interpersonal aspects of the professional tennis environment. Previous studies have examined various aspects of this environment, such as players’ perceptions of coaching effectiveness and styles [19], anticipation and decision-making processes [20] and case studies of players working with a sport psychologist [10]. More recent qualitative research has also explored professional tennis from the perspective of elite coaches [21]. However, existing research has primarily focused on discrete psychological skills, coaching processes, interpersonal dynamics, or performance-related outcomes, providing only a fragmented understanding of psychological preparation within the broader context of professional tennis. As a result, little is known about how professional tennis players themselves construct, understand, and integrate psychological preparation throughout their careers, or how they perceive its role in managing the psychological demands of professional competition. No qualitative study has examined psychological preparation as a comprehensive and evolving aspect of players’ lived experiences across different stages of a professional tennis career. The present study addresses this gap by adopting a player-centered perspective and exploring psychological preparation through professional players’ own experiences and interpretations.

Addressing this gap is particularly relevant given the distinctive psychological demands of professional tennis and the highly individualized nature of players’ competitive experiences. Rather than focusing on the effectiveness of particular mental skills or interventions, a player-centered qualitative approach can provide insight into how psychological preparation is integrated within the broader context of a professional tennis career. It can illuminate how players perceive its value, engage with psychological support, and navigate confidence, emotions, motivation, pressure, success, and failure across different stages of their careers.

Qualitative methodologies are particularly well suited to addressing this gap because they provide access to the subjective meanings, experiences, and contextual factors that shape athletes’ psychological preparation and development. Rather than evaluating the effectiveness of a particular psychological technique or intervention, a qualitative approach enables exploration of how professional players themselves conceptualize psychological preparation and understand its role within the broader context of professional tennis.

Accordingly, this qualitative study aims to examine how Czech professional-level tennis players perceive and make sense of psychological preparation across their careers. The study’s novelty lies in investigating psychological preparation as a broader and evolving component of the professional tennis experience while also considering the performance-related and psychological demands that shape players’ perspectives. While the study was not structured around a single predefined theoretical framework, the interpretation of participants’ experiences was informed by established sport psychology perspectives relevant to the patterns identified in the data, including Self-Determination Theory [5], Bandura’s self-efficacy theory [22], Hanin’s Individual Zones of Optimal Functioning (IZOF) model [23], Lazarus’s transactional perspective on stress and coping [24], and Gross’s process model of emotion regulation [25]. These perspectives were used as interpretative lenses rather than as predetermined categories for data collection or coding [26].

The research question guiding this inquiry is: How do professional tennis players interpret and conceptualize their experiences with psychological preparation, particularly in relation to emotions, motivation, and self-regulation, within the context of their professional careers? By addressing this question, the study seeks to advance understanding of the role psychological preparation plays in professional tennis and to inform both applied psychological support and future research in professional sport.

## 2. Materials and Methods

The study utilizes reflexive thematic analysis (RTA), informed by an experiential and phenomenological orientation. Epistemologically, the study adopted a contextualist position, recognizing participants’ accounts as meaningful reflections of their contextually situated subjective experiences while acknowledging that these accounts, and the knowledge generated through their analysis, are situated within particular social, cultural, and interpersonal contexts [26]. Ontologically, this approach accepts that reality is not a singular, objectively discoverable entity, but rather a multiplicity of subjective and contextually situated experiences, particularly focusing on the “lived world” of the participants [27,28,29]. The aim was to understand what participants’ experiences were like, what they mean to them, and how participants made sense of them [29]. Consistent with this experiential orientation, participants’ accounts were treated as providing insight into their subjective realities and meaning making, while themes were understood as interpretative analytic constructions generated through the researchers’ reflexive engagement with the data rather than as objectively existing entities awaiting discovery [28,30,31].

This study was informed by the diverse positionalities of the research team. The first author, a former professional tennis player (career-high WTA ranking within the top 600), held a Bachelor’s degree in psychology at the time of data collection. Psychological preparation was undervalued during her career, and seeking mental support was even stigmatized. Her dual perspective—as an athlete and client of performance psychology—provided insider insight into professional tennis. Currently active in psychological skills training with athletes and clubs, she brought valuable domain expertise. However, close attention to her personal experience was necessary to maintain critical self-reflection, especially when themes resonated strongly. Prior to the interviews, participants were informed about the first author’s background in professional tennis and psychology and were aware that the interviews were being conducted as part of academic research on psychological preparation in professional tennis.

This insider status facilitated rapport building and helped create an environment in which participants felt comfortable discussing sensitive experiences. At the same time, it may have shaped the questions asked, the accounts generated within the interview interaction, and the researchers’ subsequent interpretation of the data. Awareness of these potential influences informed ongoing reflexive practice throughout data collection and analysis.

To support reflexivity throughout the research process, the first author kept a reflexive journal, documenting thoughts, assumptions, and potential influences on data collection and interpretation. Journal entries were reviewed throughout the study and informed adjustments in interview probing, encouraged consideration of alternative interpretations during coding, and helped identify assumptions that could influence theme development. For example, reflections concerning the author’s own experiences of competitive anxiety prompted closer examination of participants’ accounts that differed from these experiences.

All interviews were conducted by the first author. Prior to data collection, the first author had no formal therapeutic or coaching relationships with the participants. She had prior training in qualitative research methods and previous experience conducting semi-structured interviews in sport-related research. As a former professional tennis player, she was familiar with the professional tennis environment and was occasionally acquainted with some participants through the tennis community.

The other authors brought an external perspective. The second author is a sports psychologist with practical experience in professional sport, specifically in a different sport than tennis, and actively focuses on qualitative research. The second author contributed to the preparation of the study and the review of relevant literature, and provided feedback throughout the research process. The third author is a methodology expert, specializing in psychological research, and contributed feedback on the manuscript and its methodological aspects. Their contributions supported critical reflection and theoretical grounding of the research. Rather than seeking an objective interpretation, differing researcher perspectives were used as analytic resources to challenge assumptions, explore alternative understandings of the data, and deepen the interpretation of participants’ experiences. The combination of insider and outsider perspectives enriched the analytic process through ongoing dialogue and reflexive engagement with the data.

The sample for this study consisted of eight Czech professional tennis players (four female and four male). For the purposes of this study, a professional tennis player was defined as an athlete actively competing in internationally sanctioned professional tournaments and holding an official ATP or WTA singles ranking at the time of data collection. Inclusion criteria were: (a) active participation in professional international tennis tournaments, (b) possession of a current ATP or WTA singles ranking, and (c) willingness to participate in an interview. Individuals who were no longer actively competing on the professional circuit were not eligible for participation. At the time of data collection, participants held ATP or WTA singles rankings between 145 and 719. This ranking range reflects the final sample and was not used as an a priori inclusion criterion. Participants represented a range of ATP/WTA ranking positions, including athletes competing at higher international levels (see Table 1 for participants characteristics).

A purposive sampling method was used to select participants based on specific criteria. Purposive sampling is particularly effective for studies that aim to gain in-depth insights into a particular phenomenon by selecting participants who have direct experience with the topic. It increases the richness and depth of the data [32]. It is commonly used in qualitative research, which focuses on in-depth exploration of specific groups or individuals who can provide valuable and relevant information [33]. Potential participants were identified through the first author’s professional network within the Czech tennis community and publicly available ATP/WTA ranking information. Participants were initially contacted via email, which clearly outlined the purpose of the study and the conditions of participation. Twelve professional tennis players meeting the inclusion criteria were contacted. Eight agreed to participate, while four did not participate (two declined due to time constraints, and two did not respond to the invitation). No substantial differences in observable characteristics (e.g., age, competitive level, or gender) were identified between participants and non-participants. The final sample size of eight participants was guided by the concept of information power, which suggests that the more relevant information a sample holds for the study aim, the fewer participants are required [34].

Data were collected through individual semi-structured interviews. This interview format allowed for flexible, in-depth exploration of participants’ perspectives [35]. The interview guide was developed based on the literature on psychological preparation in sport, qualitative interviewing, and reflexive thematic analysis. The guide was organized around broad experiential topics to encourage participants to reflect on their sporting careers, experiences with psychological preparation, sources of support, and the psychological aspects of competition before, during, and after matches. Additional open-ended prompts were used to facilitate discussion and enable participants to elaborate on personally meaningful experiences. The flexible structure of the interview allowed exploration of emerging topics while maintaining alignment with the study aims [35]. The complete interview guide is provided in Supplementary Material S1. The interview guide was pilot-tested with one tennis player prior to data collection to assess the clarity and relevance of the questions. Minor wording adjustments were subsequently made to improve question clarity. Data collection took place between January and April 2023. Seven interviews were conducted via Google Meets, while one interview was conducted face-to-face in Prague. The interviews lasted between 68 and 111 min. Participants were interviewed from private locations of their choosing, and no other individuals were present during the interviews. All interviews were conducted in Czech by the first author. No repeat interviews were conducted. Field notes documenting contextual observations and initial reflections were recorded immediately following each interview. While differences in interview mode may have influenced rapport and interaction, both interview modes yielded data relevant to the research aim. Interviews were transcribed verbatim in Czech and coded in the original language. Quotations were translated from Czech into English by the first author after the analysis had been completed. To preserve meaning and translation accuracy, the translated extracts were subsequently reviewed and discussed within the research team. All interviews were transcribed verbatim.

Data were analyzed using Braun and Clarke’s six-phase reflexive thematic analysis (RTA) [26,28]. The analysis was conducted manually without the use of qualitative data analysis software. Although the analysis was primarily inductive and grounded in participants’ accounts, it was also theoretically informed by concepts from sport psychology. Accordingly, the study adopted a theoretically informed inductive approach, in which codes and initial theme development were generated through close engagement with the data rather than derived from predefined theoretical constructs. Following the development of patterns of shared meaning, established sport psychology perspectives—including Self-Determination Theory [5], Bandura’s self-efficacy theory [22], Hanin’s Individual Zones of Optimal Functioning (IZOF) model [23], Lazarus’s transactional perspective on stress and coping [24], and Gross’s process model of emotion regulation [25]—were used as interpretative lenses to deepen understanding of motivational, confidence-related, stress-related, and emotional processes reflected in participants’ accounts. These theoretical perspectives informed the interpretation and contextualization of the findings rather than the generation of codes or themes.

The analytic process was iterative, reflexive and interpretative. The first author immersed herself in the dataset through repeated reading of all interview transcripts and generated initial observations and analytic notes. The entire dataset was coded, with attention to both explicit (semantic) and underlying (latent) meanings relevant to the research question. Initial codes were generated through close engagement with the data and were continuously revised, refined, merged, or discarded as understanding of the dataset deepened. Codes captured meaningful aspects of participants’ experiences and served as interpretative tools for exploring patterns of shared meaning across the dataset. Following initial coding, related codes were organized into provisional clusters that functioned as an analytical tool for exploring broader patterns of meaning. These clusters were visually mapped and reviewed in relation to both the coded extracts and the dataset. Through ongoing reflexive interpretation, the researcher developed patterns of shared meaning across and within these clusters, which were subsequently developed into candidate themes and refined into final themes organized around a central organizing concept.

For example, extracts describing shaking before matches, feelings of helplessness, fear of disappointing others, uncertainty about future career opportunities, and loss of enjoyment in tennis were initially coded as “competitive anxiety”, “helplessness under pressure”, “fear of failing others”, “career uncertainty”, and “loss of enjoyment”. These codes were subsequently organized around a developing pattern concerning the psychological burden associated with professional tennis. Through iterative engagement with the dataset, this pattern evolved into the final theme, “Managing the Psychological Demands of Professional Tennis”, which captured how athletes experienced, navigated, and adapted to the emotional pressures and uncertainties associated with professional-level competition. Theme development was supported by two reflexive discussions among the research team. These discussions provided opportunities to challenge interpretations, consider alternative understandings, and deepen reflexive engagement with the data.

Throughout the process, analytic memos were maintained to record emerging interpretations, and reflections on the researchers’ assumptions and positionality. The final analysis and written account were prepared by the first and second authors and subsequently reviewed by all authors. This process enabled the development of themes that captured shared patterns of meaning while remaining grounded in participants’ accounts. Quality was considered in line with recommendations for reflexive thematic analysis [28]. Throughout the study, attention was paid to methodological coherence between the research question, data generation, analytic approach, and interpretation. Reflexive memo writing and ongoing critical engagement with developing interpretations supported interpretative rigor and transparency throughout the analytic process.

The study was conducted in accordance with the principles of the Declaration of Helsinki. The research design, including both the academic and ethical aspects of the proposed research, was reviewed and approved by the Scientific Board of the Prague College of Psychosocial Studies, Prague, Czech Republic, on 19 July 2022, prior to data collection. As part of this approval process, the Scientific Board reviewed the planned procedures concerning informed consent, confidentiality and data protection, participant welfare and potential risks associated with participation, as well as the inclusion of a participant under the age of 18. The Scientific Board concluded that the proposed research procedures met the institution’s ethical requirements for Master’s thesis research and approved the project for implementation. Prior to the interviews, participants were informed about the aims of the study, research procedures, data handling, and confidentiality measures. Participants were informed that they could discontinue the interview at any time without providing a reason. Given the potentially sensitive nature of the topics discussed, participants were also informed that information about available psychological support services could be provided should they experience psychological distress during or following the interview.

Written informed consent was obtained from all participants before participation, in accordance with the General Data Protection Regulation (GDPR) and relevant Czech legislation (Act No. 110/2019 Coll. and Act No. 89/2012 Coll.). Participants additionally provided verbal confirmation of their willingness to participate at the beginning of the audio-recorded interview. One participant was 17 years old at the time of data collection. In accordance with ethical requirements, written informed consent was obtained from the participant’s parent, and assent was obtained from the participant prior to participation. All interviews were transcribed verbatim and anonymized. Participants were provided with their interview transcripts and were given the opportunity to review them for accuracy and to request corrections or clarifications where necessary. No participant feedback on the researchers’ interpretations or themes was sought. Identifying information was replaced with pseudonyms, and all data were coded prior to analysis. Given the small and potentially identifiable population of Czech ATP/WTA-ranked players, special attention was paid to minimizing the risk of deductive disclosure. Pseudonyms, age ranges, and aggregated participant characteristics were used to reduce the likelihood of participant identification, including for the participant aged 17 years. English-language pseudonyms were selected rather than Czech names because the study was reported in English for an international readership and because non-culturally specific pseudonyms further supported participant anonymity within a relatively small and potentially identifiable population of professional Czech tennis players. Potentially identifying characteristics were aggregated or omitted in the manuscript, and quotations were carefully reviewed to protect participant privacy while preserving their analytical value. The study adhered to established ethical standards for qualitative research, prioritizing participant autonomy, privacy, confidentiality, and data protection.

## 3. Results

To explore how tennis players perceive psychological preparation, reflexive thematic analysis generated three themes and seven subthemes describing players’ experiences with psychological preparation and mental states during competition (see Table 2 for Summary of Themes and Subthemes).

### 3.1. Psychological Preparation Is Valued but Rarely Fully Embraced

#### 3.1.1. Psychological Preparation: Important but Undervalued

Participants highlighted the difficulty of managing sudden shifts in match development, which required quick reactions and strong mental resilience. As Grace noted: “It’s important to be prepared and react quickly to those fast changes in the game.” Participants described tennis as involving frequent failures that players had to learn to manage. As Grace mentioned: “You can’t win every match, so you can’t let it get you down; you just have to keep going.” Financial and external pressures could take a toll on players’ mental states, as Sophia explained, “I kept thinking: What happens when the money runs out? I was always worried.” Taken together, these accounts suggested that participants perceived psychological resilience as essential for coping with the demands, uncertainties, and emotional fluctuations associated with competitive tennis. As Olivia shared, “The individual must be extremely mentally resilient, you have to deal with intense mental pressure that can shift from one second to the next during a match. You need to be able to respond well to whatever situation comes up and handle it with a clear head—and that’s not always easy.” Although participants recognized the importance of psychological preparation, they admitted to neglecting it or approaching it informally. Michael highlighted the lack of structured mental coaching: “I think there aren’t many mental coaches here who really focus on psychological preparation, and when someone’s supposed to do it on their own at home, it’s easy to skip—there’s always something else to do instead. But if it were part of the court routine, like having it scheduled—say 10 min or half an hour—where everyone does whatever works for them, it would be better for everyone.” Michael’s account suggested that psychological preparation was often viewed as an individual responsibility rather than an integrated part of athlete development. Despite acknowledging its importance, athletes struggled to prioritize mental skills training in environments where it was not formally embedded within daily routines. This appeared to contribute to psychological preparation being perceived as optional rather than as a routine component of performance development. Players reported initially rejecting psychological training but later came to view it as indispensable. Olivia recommended starting mental preparation early, just like physical conditioning. However, the challenges of seeking professional help, such as fears of opening or previous negative experiences, were also noted. These narratives suggested that athletes’ attitudes towards psychological support evolved over time, with appreciation often emerging only after direct experiences of the psychological demands associated with professional tennis.

Players were talking about consciously engaging in psychological preparation, while others described using psychological techniques spontaneously without explicitly identifying them as psychological preparation. William explained, “Most of this psychological work was done by tennis coaches. They teach you how to deal with stress yourself.” This suggested that psychological development frequently occurred outside formal mental skills programs and was instead embedded within everyday coaching practices. For athletes, coaches occupied a dual role, providing both performance guidance and emotional support. The significance of these relationships was reflected in James’s statement: “The way I opened up to my coach, I probably won’t open up to anyone else.” This highlighted the importance of trust within the coach–athlete relationship and suggested that athletes appeared more willing to discuss psychological concerns with a trusted coach than through formal psychological support pathways. Psychological support therefore appeared to depend not only on access to expertise but also on the quality of interpersonal relationships. Additionally, players learned psychological techniques from social media or by observing professional athletes. Sophia stressed the need for a tailored approach, noting, “Every person is unique and should find what works for them.” Taken together, participants’ accounts suggested that psychological preparation was largely informal, relational, and self-constructed, rather than systematically embedded within their developmental pathways as professional tennis players.

#### 3.1.2. Readiness for Psychological Preparation Must Come from Within

Participants emphasized that effective psychological preparation required personal readiness and internally driven engagement. Rather than rejecting psychological support itself, athletes described that its effectiveness depended on their willingness to actively participate in the process. Psychological preparation was often perceived as less beneficial when initiated primarily by parents, coaches, or other external influences. Emily recalled her first experiences with psychological preparation, “I was too young for it, so I couldn’t have a positive opinion about it. I didn’t understand what was expected of me. They sent me there too early; I was still too much of a child. You have to come to it on your own.” Similarly, James noted that he only embraced psychological preparation when he felt ready: “When I think about it, I realize that I didn’t listen to him [the psychologist]. I wasn’t ready to accept these things. I wasn’t interested. I didn’t think it was that important. A big part of it was definitely my mindset. I didn’t seek out the psychologist myself; it probably came from my parents or the coach because they could see that something was wrong with me.”

Other participants echoed this view, suggesting that engagement with psychological preparation was closely linked to athletes’ readiness for self-reflection and change. Sophia explained that although psychological support was available to her, she never felt a sufficient personal need to seek professional help and doubted that she would have been able to openly discuss her experiences with a psychologist. Daniel likewise argued that psychological preparation only works when athletes genuinely want to engage with it themselves, noting that external pressure rarely leads to sustained involvement.

These accounts suggested that psychological preparation was viewed not as something that could be imposed externally but as a self-directed process requiring personal readiness, openness, and active commitment from the athlete. Participants repeatedly emphasized that psychological support became meaningful only when they themselves recognized its value and were prepared to engage with it. Psychological readiness therefore appeared to play an important role in how participants engaged with and perceived the value of psychological preparation.

### 3.2. Finding and Regaining Psychological Control During Competition

#### 3.2.1. Confidence and Momentum as a Sense of Control

Participants described maintaining peak performance as requiring a combination of psychological resilience and emotional regulation throughout a match. Players perceived momentum as an important psychological experience that influenced how they felt and performed during competition. Rather than describing momentum as an objectively measurable factor, participants spoke about it as a subjective sense of confidence, control, and psychological ease that emerged during successful periods of play. Participants mentioned that being on a winning streak created a sense of invincibility, which they perceived as enhancing their mental state. As Olivia recalled, “When I was on a winning streak, I won a tournament, and it felt like I didn’t need to sleep or eat. I even had a phase where I won 13 matches in a row, and I would go to the court and I knew that if I did everything I was supposed to, I just wouldn’t lose.” William also shared his experience, describing the winning streak as one of the best psychological experiences, “The winning streak is one of the best things. It’s the most intense psychological experience I’ve ever had. Every time I won a match and went to relax, it didn’t matter if I played for 6, 8 or 3 h; I felt completely fine.” Participants commonly associated these periods of success with increased confidence and a greater sense of psychological control. However, they also perceived momentum as fragile and potentially accompanied by additional pressure to maintain performance. As Grace pointed out: “When you win and are on a winning streak, of course, you feel better, but you might also put more pressure on yourself.” Within participants’ accounts, confidence was described as a central psychological resource influencing how they approached preparation and competition. Players emphasized the importance of trying new situations to build confidence, while others highlighted the significance of having a “killer instinct”—the ability to perform better under pressure.

The balance between confidence and arrogance was another key point, with Michael noting: “I try to win matches. [laughs] I try to do it through determination, to win through hard work, and then the healthier self-confidence always comes.” During tough times, players remind themselves of past successes to regain focus, as Emily shared: “I’ve managed to turn around so many difficult situations during a single tournament, and even now, when I’m losing, I go back to that and remind myself that I can turn it around.” Similarly, Grace stressed the importance of maintaining self-belief, even when things are not going well: “In a game, I believe in myself, even when things aren’t going well. That’s where realism comes in, knowing what I can do and that it’s never too late to show it in a match.” William also acknowledged that self-confidence can sometimes be “faked”: “It’s really easy to fake it. I think it works. You can even fool yourself with your behavior.” Together, these accounts suggested that confidence was not experienced as a fixed psychological state but as something participants actively sought to build, restore, or maintain during competition. Previous successes, deliberate self-belief, and behavioral expressions of confidence appeared to serve as resources through which players attempted to regain a sense of psychological control.

#### 3.2.2. Regulating Arousal and Emotion Before and During Matches

Additionally, pre-match routines and in-match emotional management were viewed as critical components of a player’s mental readiness. Participants described using a range of psychological strategies to prepare for competition and regulate emotions. Some of these practices, such as visualization, breathing exercises, and self-talk, were reported as techniques learned through previous exposure to sport psychology, coaches, or other educational resources. Others appeared to emerge organically through athletes’ own competitive experiences and personal preferences. Players also described developing personalized pre-match routines to cope with the uncertainty inherent in tournament tennis. These routines included preparing equipment, following a specific warm-up routine, listening to familiar music, or engaging in rituals that participants perceived as helping them maintain psychological stability. As Olivia explained: “I have routines and rituals that I follow, and they calm me down… I want to sit down and feel like I’m alone, I need peace.” While William described routines as optional, other participants described relying on them to support focus and peace of mind before a match. Participants also described seeking solitude and limiting distractions, for example by turning off their phones.

Players mentioned that they incorporated strategic mental practices, such as analyzing their opponent’s strengths and weaknesses or visualizing optimal performance scenarios. Michael shared his flexible approach to mental preparation, stating: “When I get into the zone [through breathing exercises], I either try to visualize something or tell myself what I want to do or how I want the training session or match to go, including certain moments. Especially during the match I go through the game plan, how I want to play, how the opponent plays, so I can see it clearly.” Participants described pre-match nervousness as part of their competitive experiences. In their accounts, a moderate level of nervousness was perceived as beneficial for readiness to compete, whereas excessive nervousness was perceived as detrimental to performance. James explained, “I need to feel the adrenaline… the butterflies in my stomach… but it shouldn’t be too much.” Players often reported experiencing physical symptoms of nervousness, such as increased heart rate or trembling. To manage these, they used a mix of cognitive strategies (like rationalization and acceptance), physical methods (such as breathing exercises), and managing body language.

Emotional regulation was viewed by participants as critical not only in moments of failure but also when playing well, as players like William emphasized: “I don’t focus on what’s not working; I focus on what I can do to make it work.” In fact, even when performing well, players reported experiencing anxiety about maintaining form. As Michael shared: “Even when things go well, sometimes I still have unnecessarily negative thoughts”. Players acknowledged that they also engaged in emotional expression to relieve tension, such as swearing or reminding themselves to stay focused. Sophia admitted: “I swear or tell myself to stay focused and keep playing.” Participants differed in how they perceived the usefulness of these responses, with some viewing emotional expression as a way of releasing tension and others preferring a calmer approach. James, for example, noted that acknowledging emotions is a key step in regulation: “Once I realize what’s happening to me at that moment, I can work with it.” Taken together, participants’ accounts suggested that emotion regulation was not experienced as the elimination of nervousness or unpleasant emotions, but as an ongoing process of recognizing, managing, and responding to changing emotional states during competition. The strategies used varied considerably between players, reinforcing the individualized nature of psychological regulation within competitive tennis.

#### 3.2.3. Creating Distance to Process Success and Failure

Participants described processing match outcomes as involving two distinct phases: an immediate emotional reaction followed by later reflection and evaluation. Players noted that losses often triggered emotions like anger, regret, and disappointment, especially in close matches where a few key mistakes could decide the outcome. Participants explained that their initial responses to defeat were often highly emotional and characterized by frustration, sadness, or self-criticism. Nevertheless, players acknowledged that losing was a natural part of the sport. William remarked: “Every athlete knows you have to lose to win. You can’t win all your life, and if you do, hats off, you’re good.” While the immediate aftermath of a loss was often dominated by emotion, participants emphasized the importance of creating distance from these initial reactions before evaluating their performance. Dealing with defeat was often perceived as easier when players felt confident that they had given their best effort. Participants also felt that experience at higher levels of competition helped them cope more effectively with losses. Reflecting on defeats in a measured way, rather than immediately and emotionally, was described as an important part of making sense of both success and failure. Sophia shared: “I don’t think you should analyze the match immediately… an hour later I wouldn’t have said the same thing.” In contrast, participants associated victories with pleasant emotions, although some wins were described as feeling routine, particularly when the opponent was perceived as weaker. However, participants suggested that pleasant emotions associated with winning were often followed by more reflective evaluation of their performance. Grace stressed: “Even when things go well, it’s important to identify areas for improvement”. As Daniel stated: “When you win, training is easier, the whole process is more pleasant.” Participants described delayed reflection as allowing them to evaluate both wins and losses with greater distance from their immediate emotional reactions.

### 3.3. Managing the Psychological Demands of Professional Tennis

#### 3.3.1. Experiencing the Psychological Burden of Professional Tennis

Participants described periods of substantial psychological strain associated with professional tennis. These experiences were often linked to injury, prolonged periods of poor performance, uncertainty about career progression, financial pressure, or the fear of failing to meet personal and external expectations. For some athletes, tennis became closely tied to their sense of self-worth, making setbacks particularly difficult to manage.

Participants reported a range of emotional and physiological responses, including anxiety, helplessness, emotional exhaustion, and loss of enjoyment. In more severe cases, psychological distress manifested through somatic symptoms such as nausea, vomiting, shaking, episodes described by participants as panic attacks and feelings of paralysis during competition. Sophia described a prolonged period in which these reactions became particularly intense: “I got into such a bad mental state—honestly, it was a mess. It only happened in singles, not in doubles. I’d walk onto the court with shaking hands, I’d feel sick, sometimes even throw up—it was awful. It went on for almost half a year. I was struggling, stuck in it, and it was really tough.” Similar experiences were reflected in Emily’s account: “I couldn’t breathe, I was crying and shaking. I didn’t know what to do.” These accounts illustrated how participants experienced psychological strain not only emotionally but also through pronounced physical reactions during competitions. For other participants, psychological strain was experienced as an inability to perform in accordance with their perceived capabilities. Despite recognizing that they possessed the necessary technical and physical abilities, they described being unable to reproduce them under pressure. James captured this discrepancy between perceived ability and competitive performance: “I knew I could play, but I was completely paralyzed. I couldn’t produce even a fraction of what I knew I was capable of.” The psychological burden of these experiences also extended beyond individual matches. Fear of failure, social comparison, and uncertainty about future career opportunities contributed to participants’ accounts of sustained pressure, while repeated difficulties could affect their relationship with tennis itself. As Grace described, “Tennis became an enemy. I didn’t even want to go to practice anymore.” Her account illustrated how periods of psychological strain could coincide with a loss of enjoyment and a sense of disconnection from the sport.

#### 3.3.2. Reframing Success Through Support, Perspective and Growth

Although participants described substantial psychological struggles, they also reported developing strategies and perspectives that helped them adapt to the emotional demands of professional tennis. Sources of support included coaches, family members, partners, teammates, professional psychologists, and personal reflection gained through experience. Athletes emphasized that periods of injury, forced breaks, or time away from competition enabled them to reconsider their priorities and develop a broader perspective on life beyond tennis. Participants associated this broader perspective with reduced performance pressure and greater emotional stability. James described this shift simply: “I realized that tennis wasn’t everything anymore. Life goes on.” For some participants, this reframing involved redirecting attention toward other meaningful aspects of their lives or careers. Sophia described doubles as providing an alternative competitive focus: “The doubles became my priority and that was what helped me get out of it.” Similarly, Grace referred to education as an important source of perspective beyond her tennis career: “I realized that even if tennis doesn’t work out, I still have my studies.” Together, these accounts suggested that developing sources of meaning and identity beyond immediate competitive outcomes helped participants place tennis within a broader life context. Athletes also described drawing on self-talk, breathing strategies, acceptance, and lessons learned through experience. Rather than eliminating adversity, participants perceived these approaches as helping them tolerate uncertainty, maintain perspective, and regain a sense of control. Sophia described this process as recognizing her internal state and deliberately returning to a more helpful response: “I simply needed to realize what was happening and return to what I knew was the right reaction.”

Psychological adaptation was also reflected in participants’ descriptions of moments in which they experienced complete absorption in performance. These experiences were characterized by a heightened sense of presence, control, and enjoyment. Olivia described such an experience: “It felt like the ball was coming at me in slow motion… I felt totally present, like I had everything under control.” Her account illustrated how participants’ experiences of professional tennis encompassed not only psychological strain but also moments of psychological ease, control, and renewed connection with performance.

## 4. Discussion

This study provides an in-depth analysis of the psychological preparation of professional Czech tennis players. Despite evidence indicating beneficial effects of mental skills training in racket sports [9], participants predominantly described relying on their own coping strategies rather than systematically implemented psychological preparation. Participants frequently described coaches as important sources of informal psychological and emotional support, which is consistent with research showing that elite tennis players value coach–athlete relationships characterized by trust, emotional support, positive feedback, and open communication [19]. The findings therefore suggest that greater attention to systematic psychological preparation may be beneficial within professional tennis environments [14]. Participants’ experiences indicate that coaches and mental health professionals may have complementary roles in supporting athletes’ psychological development. While coaches may contribute to psychologically supportive training environments and facilitate access to appropriate support, they should not be regarded as substitutes for qualified mental health professionals. Participants’ experiences further suggest that sport psychologists may benefit from considering athletes’ individual characteristics, preferences, and personal experiences when providing psychological support. This is consistent with evidence indicating that elite athletes employ diverse coping strategies and that their effectiveness may vary according to individual and situational factors [36]. Similarly, Moutaraji et al. [37] reported differences in mental toughness and coping skills according to sport type and competitive level, further highlighting the importance of considering athletes’ individual and contextual characteristics when providing psychological support. Collectively, these findings point to the potential value of tailoring psychological support to athletes’ individual characteristics and the specific demands of competitive situations. They further suggest that early exposure to psychological preparation may be beneficial, while emphasizing the importance of adapting such support to athletes’ personal needs, preferences, and readiness.

A key finding of this study is the importance of psychological readiness in engaging with psychological preparation. Participants consistently emphasized that the perceived value of psychological support depended not only on its availability but also on their willingness to actively engage in the process. Athletes described that psychological preparation was less beneficial when initiated primarily by parents, coaches, or other external influences and became meaningful only when they personally recognized its relevance and were ready to participate. These findings are consistent with Self-Determination Theory, which proposes that autonomous motivation and self-endorsed behaviors promote greater engagement and persistence in personally meaningful activities [5]. The findings suggest that the availability of psychological support alone may not be sufficient for meaningful involvement in the psychological preparation process. Similarly, evidence-informed frameworks in elite sport emphasize that psychological and mental health support should be person-centered, autonomy-supportive, and embedded within a broader organizational culture that promotes psychological wellbeing and help-seeking [18]. These findings suggest that athletes may engage more meaningfully with psychological preparation when they are supported in developing readiness, autonomy, and personal ownership of the process. Future research could further explore the role of psychological readiness in athletes’ engagement with psychological support and mental skills development in professional sport.

Another theme generated through the analysis concerned momentum and self-confidence. Participants perceived winning streaks as contributing to increased self-belief and a stronger sense of control over performance. Participants described adopting a ‘fake it till you make it’ approach, portraying confidence even when they did not fully experience it themselves. This can be understood through the lens of Bandura’s concept of self-efficacy [22], as beliefs about one’s ability to succeed are considered important in determining how individuals approach challenging situations. However, this connection is a theoretical interpretation of the participants’ accounts rather than direct evidence of a causal mechanism. Participants’ accounts suggested that confidence was perceived as an important psychological resource that shaped their approach to demanding competitive situations. This interpretation is consistent with meta-analytic evidence demonstrating an overall positive association between self-confidence and sport performance, while also indicating substantial variability in the strength of this relationship across studies [8]. At the same time, confidence appeared to be both beneficial and fragile, particularly during periods of success, when concerns about maintaining performance could arise. This finding is broadly consistent with previous research showing that even world-class athletes can experience disruptions in sport confidence and highlighting the importance of interventions aimed at protecting and maintaining confidence under competitive pressure [38]. The findings suggest that helping athletes to navigate fluctuations in confidence and the pressures associated with competitive success may be an important part of psychological preparation. Participants’ accounts highlight the complexity of confidence in professional sport and suggest that further research is needed to better understand how athletes experience and manage fluctuations in self-confidence.

Participants also highlighted the importance of psychological preparation before competition. Pre-match routines, visualization, breathing exercises, self-talk, and game planning were described as strategies that helped athletes create a sense of readiness and control in the inherently unpredictable context of tournament tennis. These findings are consistent with previous research highlighting the role of pre-performance routines and psychological skills in preparing athletes for competition and regulating arousal [6,12]. Mental skills support has also been applied within professional tennis, with case-study evidence illustrating the contextual implementation of psychological skills as part of competitive preparation [10]. The use of visualization and breathing described by participants is also supported by recent tennis-specific experimental research showing that these strategies, particularly when combined, can improve specific aspects of serve performance, including accuracy, speed, and efficiency [39]. A key finding of this study is that emotion regulation is an ongoing process throughout a match rather than a task limited to moments of failure or crisis. This dynamic view is consistent with Lazarus’s transactional perspective, which conceptualizes emotions and coping as ongoing processes shaped by individuals’ appraisals of changing situational demands [24]. Participants emphasized the importance of managing pleasant and unpleasant emotions to stay ‘in the zone’ and maintain performance. Participants also described pre-match nervousness as a common aspect of competition, often perceiving moderate levels of nervousness as beneficial for performance readiness, whereas excessive nervousness was viewed as potentially detrimental. This finding is consistent with Hanin’s IZOF model, which emphasizes that the functional effects of emotional states depend on their individually optimal intensity and quality rather than simply on whether they are experienced as pleasant or unpleasant [23]. Participants described using a variety of emotion regulation strategies, including cognitive reframing, emotional expression (venting), and suppression. These strategies appeared to serve different functions and varied across situations and individuals. This variability is consistent with contemporary emotion-regulation perspectives emphasizing that the effectiveness and consequences of regulatory strategies depend on how and in what context they are used [25]. However, participants reported feeling emotionally depleted following repeated setbacks. These accounts may reflect the potential costs of sustained emotion-regulatory effort, a phenomenon described in the sport literature as regulation depletion [40]. Their experiences also raise questions about the potential costs associated with relying heavily on suppression as an emotion-regulation strategy [25]. Importantly, recent research emphasizes that emotion regulation in sport extends beyond individual processes and is embedded within broader social and cultural contexts [4]. Participants frequently cited coaches as important sources of interpersonal support, further highlighting the collaborative nature of emotion regulation. Taken together, these findings point to the potential relevance of considering emotional, motivational, cognitive, and behavioral aspects of self-regulation within psychological preparation rather than viewing individual mental skills in isolation [41]. Overall, the findings suggest that psychological preparation and emotion regulation are closely interconnected processes that may help athletes navigate the uncertainties and psychological demands of competitive tennis.

The analysis highlighted the importance participants attributed to reflecting on matches, regardless of the outcome. Participants described processing match outcomes as involving an initial emotional response followed by more deliberate reflection and evaluation. Athletes emphasized that creating distance from their immediate emotional reactions was important before attempting to analyze their performance. Players described how reflecting on their performance helped them to understand both successes and failures. This pattern suggests that reflection may be facilitated when athletes move beyond their initial emotional responses and engage in a more balanced evaluation of their performance. Research on post-performance self-regulation suggests that self-monitoring, regulation of cognition and emotion, and psychological detachment may support athletes’ recovery by helping them identify recovery needs and restore cognitive and psychological resources following competition [42]. These processes may also be relevant to reflective practice by facilitating greater psychological distance from immediate emotional reactions. Consistent with this perspective, participants viewed reflection as an important means of making sense of both success and failure, regardless of match outcome. Overall, the findings highlight reflective practice as a potentially important component of how athletes process competitive experiences and learn from both victories and defeats.

Athletes in this study vividly described the substantial psychological strain they experienced as a result of playing professional tennis. They linked these experiences to injury, prolonged poor performance, uncertainty about career progression, financial pressures and worries about meeting personal and external expectations. For some athletes, tennis was closely intertwined with their sense of identity and self-worth, making setbacks particularly challenging. These accounts are consistent with previous research [1] highlighting the substantial psychological demands of competitive tennis, and with broader evidence showing that elite athletes are exposed to multiple sport-related and psychosocial stressors that may contribute to psychological distress [15,16]. The combination of chronic stress, high expectations, financial and interpersonal pressures, and insufficient recovery has also been identified as a factor in the development of athlete burnout [43]. The emotional exhaustion and loss of enjoyment described by participants resemble experiences reported in the athlete burnout literature [43]. Participants also described a range of physiological manifestations of psychological distress, including trembling, nausea, difficulty breathing, and experiences they characterized as panic attacks. Although the present study did not assess clinical symptoms, these accounts illustrate the extent to which psychological strain may be experienced not only emotionally but also physically. These accounts can be interpreted through transactional perspectives on stress and coping, in which distress may occur when perceived demands exceed available coping resources [24].

At the same time, participants’ accounts demonstrated that adaptation to these challenges was often facilitated through social support, personal reflection, and the development of broader life perspectives. Athletes frequently identified coaches, family members, partners, teammates, and, in some cases, sport psychologists as important sources of support. These findings are consistent with research suggesting that social support may help reduce stress and vulnerability to athlete burnout [43], while supportive and psychologically safe interpersonal environments may also contribute to athletes’ psychological well-being [17,18].

A particularly notable finding was participants’ description of how injury, time away from competition, or changes in life priorities contributed to a broader perspective beyond tennis. Athletes associated recognizing alternative sources of identity, such as education, relationships, or other sporting roles, with reduced performance-related pressure and greater emotional stability. This aligns with previous research highlighting the potential importance of supportive relationships, activities outside sport, and maintaining a broader life perspective beyond the athletic role for athletes’ psychological well-being, particularly during periods of injury and career transition [43,44]. Participants also described how they relied on acceptance, self-talk, breathing strategies and lessons learned through experience to help them navigate challenging situations. Rather than eliminating adversity, these approaches seemed to enable athletes to tolerate uncertainty, maintain perspective and stay involved in the sport. Taken together, these findings suggest that adaptation to the psychological demands of professional tennis is shaped by both individual coping resources and supportive relationships, as well as the ability to develop meaningful perspectives beyond competitive performance. This is consistent with research emphasizing the dynamic interplay between athletes’ personal coping resources and supportive social environments in promoting mental health in elite sports [18,44]. Further research could explore how these factors contribute to long-term resilience and well-being in professional tennis players.

While this study provides rich qualitative insights into the psychological experiences of professional tennis players, several limitations should be acknowledged. First, the study employed purposive sampling, and participation was voluntary. Consequently, athletes willing to discuss psychological experiences may differ systematically from those who declined participation. Participants may have been more open to psychological topics, more reflective, or more willing to disclose personal experiences, which may have influenced the range and nature of perspectives represented in the findings.

Second, the sample consisted exclusively of Czech professional tennis players. As athletes’ experiences of psychological preparation, help-seeking, and career development are embedded within specific cultural and organizational contexts, caution is warranted when considering the transferability of these findings to athletes from other countries, sporting systems, or cultural environments. In addition, the sample was relatively small and included players with varying competitive rankings and career trajectories. Although this diversity provided a broad range of perspectives, experiences may differ across performance levels, potentially limiting the extent to which the findings reflect the experiences of professional tennis players as a more homogeneous group.

Third, participants described experiences occurring across different stages of their careers. As a result, the findings are based on retrospective self-report and may be influenced by recall bias. Participants’ current perspectives and subsequent experiences may have shaped how earlier events were remembered and interpreted. Furthermore, the interviews were conducted in Czech, whereas the findings are reported in English. Although care was taken to preserve the meaning of participants’ accounts during translation, some nuances, cultural meanings, or contextual subtleties may have been modified or lost during the translation process.

Researcher positionality should also be considered when interpreting the findings. The first author’s background as a former professional tennis player and current sport psychology practitioner provided valuable contextual understanding and facilitated rapport with participants. At the same time, this positioning may have influenced both data generation and interpretation. Given the relatively small population of professional Czech tennis players, the first author was familiar with some participants through the professional tennis environment. Although no coaching relationship existed between the researcher and participants, prior acquaintance may have influenced rapport, disclosure, and interpretation. To critically engage with the potential influence of researcher positionality, reflexive discussions were maintained throughout the research process, and interpretations were reviewed within the research team. In addition, the interviewer’s background and perceived expertise in professional tennis may have influenced participants’ responses. Some participants may have emphasized socially desirable attitudes, experiences, or coping strategies, particularly when discussing psychological challenges and help-seeking behaviors. Consequently, social desirability effects cannot be ruled out.

Another limitation relates to data collection procedures. Most interviews were conducted online, while one interview took place face-to-face. Differences in interview modality may have influenced rapport, disclosure, and communication dynamics, potentially affecting the depth and nature of the data collected. Furthermore, although participants were given the opportunity to review and approve their interview transcripts, analytic member reflection was not conducted. Participants were therefore not invited to engage with or reflect on the researchers’ final thematic interpretations. Consistent with reflexive thematic analysis, the themes presented represent the researchers’ situated interpretations of patterns of shared meaning across participants’ accounts rather than findings requiring participant verification. Finally, the time elapsed between data collection and publication should be considered, as athletes’ circumstances, competitive environments, and perspectives may have evolved during this period. Although participants frequently described limited access to structured psychological support at the time of data collection, the professional tennis environment may have evolved since then. Therefore, these findings should not be interpreted as evidence that such limited provision necessarily characterizes the current professional tennis context.

Importantly, as a qualitative study, the present research was not designed to estimate prevalence. Although participants described panic-like experiences, emotional exhaustion, or substantial psychological distress, these findings cannot be used to determine how common such experiences are among Czech or international professional tennis players. Similarly, the study cannot establish whether psychological preparation or psychological interventions improve athletic performance or mental health outcomes. Future research could include athletes from various sports to broaden understanding of psychological preparation and emotion regulation in competitive settings. Quantitative studies may further examine the prevalence of the experiences identified in the present study, while intervention-based research could evaluate the effects of specific psychological preparation strategies on performance and mental health outcomes.

In conclusion, this study highlights the complex interplay of individual, relational, and contextual factors shaping the psychological experiences of eight Czech professional tennis players. Participants perceived psychological preparation as important for navigating the demands of professional tennis yet often engaged with it informally and only when personally ready to do so. Their accounts further emphasized the role of self-confidence, perceived momentum, emotion regulation, and reflective processing of both success and failure in managing competitive demands. At the same time, participants described substantial psychological strain associated with professional tennis and associated social support, broader personal perspectives, and experiences beyond sport with adaptation and well-being. Participants’ accounts point to the potential value of considering not only individual coping processes but also the relational and organizational contexts in which athletes train and compete when providing psychological support. Further research is needed to examine these experiences across different sporting and cultural contexts and to inform context-sensitive approaches to supporting athletes’ psychological well-being and development.

## Figures and Tables

**Table 1:** Participants’ Characteristics.

Code	Gender	Age Range	Professional Experience	Experience with Sport Psychologist	Alias
T01	Female	17–19	4	Yes	Emily
T02	Male	23–26	8	Yes	James
T03	Female	23–26	8	Yes	Olivia
T04	Female	23–26	9	No	Sophia
T05	Male	20–22	5	No	Daniel
T06	Male	23–26	8	Yes	Michael
T07	Male	17–19	2	No	William
T08	Female	20–22	8	No	Grace

Note: Age is reported in ranges to protect participant confidentiality and reduce the risk of deductive identification.

**Table 2:** Summary of Themes and Subthemes.

Theme	Subtheme
Psychological Preparation Is Valued but Rarely Fully Embraced	Psychological Preparation: Important but Undervalued
	Readiness for Psychological Preparation Must Come from Within
Finding and Regaining Psychological Control During Competition	Confidence and Momentum as a Sense of Control
	Regulating Arousal and Emotion Before and During Matches Creating Distance to Process Success and Failure
Managing the Psychological Demands of Professional Tennis	Experiencing the Psychological Burden of Professional Tennis Reframing Success Through Support, Perspective, and Growth

## Data Availability

The data presented in this study are not publicly available due to ethical and privacy considerations and will not be shared upon request. The study is based on qualitative interviews with a small and potentially identifiable group of professional athletes. Sharing the interview transcripts could compromise participant confidentiality, privacy, and anonymity. Data access is therefore restricted in accordance with the conditions of participant consent and ethical approval.

## Supplementary Materials

The following supporting information can be downloaded at: https://www.mdpi.com/article/10.3390/sports14100429/s1, Supplementary Material S1: Semi-structured interview guide (original Czech version); Supplementary Material S2: Semi-structured interview guide (English translation); Supplementary Material S3: COREQ Checklist [45].

## References

[1] Konstantinou G.N., Kloiber S., Blumberger D.M. (2025). The Mental Game of Tennis: A Scoping Review and the Introduction of the Resilience Racket Model. Sports.

[2] Cowden R.G. (2016). Competitive Performance Correlates of Mental Toughness in Tennis: A Preliminary Analysis. Percept. Mot. Ski..

[3] Öztürker C., Şahan A., Erman K.A. (2025). Impact of Mental Fatigue on Tennis Players’ Attention and Groundstroke Performance. Front. Psychol..

[4] Tamminen K.A., Wolf S.A., Dunn R., Bissett J.E. (2024). A Review of the Interpersonal Experience, Expression, and Regulation of Emotions in Sport. Int. Rev. Sport Exerc. Psychol..

[5] Ryan R.M., Deci E.L. (2000). Self-Determination Theory and the Facilitation of Intrinsic Motivation, Social Development, and Well-Being. Am. Psychol..

[6] Gould D., Udry E. (1994). Psychological Skills for Enhancing Performance: Arousal Regulation Strategies. Med. Sci. Sports Exerc..

[7] Liu Y.-C., Wang M.-Y., Hsu C.-Y. (2018). Competition Field Perceptions of Table-Tennis Athletes and Their Performance. J. Hum. Kinet..

[8] Jekauc D., Fiedler J., Wunsch K., Muelberger L., Burkart D., Kilgus A., Fritsch J. (2025). The Effect of Self-Confidence on Performance in Sports: A Meta-Analysis and Narrative Review. Int. Rev. Sport Exerc. Psychol..

[9] Cece V., Guillet-Descas E., Martinent G. (2020). Mental Training Program in Racket Sports: A Systematic Review. Int. J. Racket Sports Sci..

[10] Mathers J.F. (2017). Professional Tennis on the ATP Tour: A Case Study of Mental Skills Support. Sport Psychol..

[11] Ducrocq E., Wilson M., Vine S.J., Derakshan N. (2016). Training Attentional Control Improves Cognitive and Motor Task Performance. J. Sport Exerc. Psychol..

[12] Cotterill S.T. (2010). Pre-Performance Routines in Sport: Current Understanding and Future Directions. Int. Rev. Sport Exerc. Psychol..

[13] Birrer D., Morgan G. (2010). Psychological Skills Training as a Way to Enhance an Athlete’s Performance in High-Intensity Sports. Scand. J. Med. Sci. Sports.

[14] Wagstaff C.R.D., Quartiroli A. (2023). A Systems-Led Approach to Developing Psychologically Informed Environments. J. Sport Psychol. Action.

[15] Gouttebarge V., Castaldelli-Maia J.M., Gorczynski P., Hainline B., Hitchcock M.E., Kerkhoffs G.M., Rice S.M., Reardon C.L. (2019). Occurrence of Mental Health Symptoms and Disorders in Current and Former Elite Athletes: A Systematic Review and Meta-Analysis. Br. J. Sports Med..

[16] Pilkington V., Rice S., Olive L., Walton C., Purcell R. (2024). Athlete Mental Health and Wellbeing During the Transition into Elite Sport: Strategies to Prepare the System. Sports Med. Open.

[17] Rice S., Walton C.C., Pilkington V., Gwyther K., Olive L.S., Lloyd M., Kountouris A., Butterworth M., Clements M., Purcell R. (2022). Psychological Safety in Elite Sport Settings: A Psychometric Study of the Sport Psychological Safety Inventory. BMJ Open Sport Exerc. Med..

[18] Purcell R., Pilkington V., Carberry S., Reid D., Gwyther K., Hall K., Deacon A., Manon R., Walton C.C., Rice S. (2022). An Evidence-Informed Framework to Promote Mental Wellbeing in Elite Sport. Front. Psychol..

[19] Zhu Q., Bloom G.A., Xu C., Alexander-Urquhart D., Guo L., Zhu Y. (2024). Elite Tennis Players’ Perceptions of Coaching Effectiveness. Int. J. Sports Sci. Coach..

[20] Carboch J., Brenton J., Reischlova E., Kocib T. (2023). Anticipatory Information Sources of Serve and Returning of Elite Professional Tennis Players: A Qualitative Approach. Int. J. Sports Sci. Coach..

[21] Clarke H., Bloom G.A., Glaude M. (2026). Coaching on the Professional Tennis Tour: Elite Coaches’ Perceptions of Contextual Challenges and Effective Coaching Strategies. J. Appl. Sport Psychol..

[22] Bandura A. (1997). Self-Efficacy: The Exercise of Control.

[23] Hanin Y.L. (2000). Emotions in Sport.

[24] Lazarus R.S. (2006). Stress and Emotion: A New Synthesis.

[25] Gross J.J. (2015). Emotion Regulation: Current Status and Future Prospects. Psychol. Inq..

[26] Braun V., Clarke V. (2022). Thematic Analysis: A Practical Guide.

[27] Braun V., Clarke V. (2019). Reflecting on Reflexive Thematic Analysis. Qual. Res. Sport Exerc. Health.

[28] Braun V., Clarke V. (2021). Can I Use TA? Should I Use TA? Should I Not Use TA? Comparing Reflexive Thematic Analysis and Other Pattern-Based Qualitative Analytic Approaches. Couns. Psychother. Res..

[29] Willig C., Cooper H. (2012). Perspectives on the Epistemological Bases for Qualitative Research. APA Handbook of Research Methods in Psychology.

[30] Braun V., Clarke V. (2022). Conceptual and Design Thinking for Thematic Analysis. Qual. Psychol..

[31] Josselson R. (2004). The Hermeneutics of Faith and the Hermeneutics of Suspicion. Narrat. Inq..

[32] Patton M.Q. (2002). Qualitative Research and Evaluation Methods.

[33] Creswell J.W. (2013). Qualitative Inquiry and Research Design: Choosing Among Five Approaches.

[34] Malterud K., Siersma V.D., Guassora A.D. (2016). Sample Size in Qualitative Interview Studies: Guided by Information Power. Qual. Health Res..

[35] Kallio H., Pietilä A.-M., Johnson M., Kangasniemi M. (2016). Systematic Methodological Review: Developing a Framework for a Qualitative Semi-Structured Interview Guide. J. Adv. Nurs..

[36] Nuetzel B. (2023). Coping Strategies for Handling Stress and Providing Mental Health in Elite Athletes: A Systematic Review. Front. Sports Act. Living.

[37] Moutaraji I., Lotfi S., Talbi M. (2021). Mental Strength and Coping Strategy of Confined Athletes Dealing with COVID-19. Int. J. Hum. Mov. Sports Sci..

[38] Hays K., Thomas O., Maynard I., Bawden M. (2009). The Role of Confidence in World-Class Sport Performance. J. Sports Sci..

[39] Robin N., Carien R., Bonnin T., Michineau L., Dominique L. (2026). The Combination of Motor Imagery and Breathing Optimizes the Performance of the Serve in Skilled Tennis Players. Sports Health.

[40] Wergin V.V., Wolf S.A., Schwender J.T., Mallett C.J. (2025). Experienced Emotions, Emotion Regulation Strategies, and Regulation Depletion in Collective Team Collapse and Performance Recovery Situations. J. Appl. Sport Psychol..

[41] Latinjak A.T. (2025). Integrative Self-Regulation Model for Sport and Exercise: Theory and Implications for Comprehensive Training. Perform. Enhanc. Health.

[42] Balk Y.A., Englert C. (2020). Recovery Self-Regulation in Sport: Theory, Research, and Practice. Int. J. Sports Sci. Coach..

[43] Gustafsson H., Kenttä P., Hassmén M. (2011). Athlete Burnout: A Systematic Review and Future Research Directions. Int. Rev. Sport Exerc. Psychol..

[44] Küttel A., Storm L., Stambulova N., Henriksen K. (2024). Threatening and Nurturing Mental Health: Insights from Danish Elite Athletes on the Dynamic Interplay of Factors Associated with Their Mental Health. Front. Sports Act. Living.

[45] Tong A., Sainsbury P., Craig J. (2007). Consolidated Criteria for Reporting Qualitative Research (COREQ): A 32-Item Checklist for Interviews and Focus Groups. Int. J. Qual. Health Care.

