# Supplementary material for: Mental Preparation in Professional Tennis: A Qualitative Study of Czech Players’ Experiences
Source: Sports (Basel). 2026 Oct 3;14(10):429. doi: 10.3390/sports14100429 (PMC13709374; doi:10.3390/sports14100429)
Supplement: Supplementary file 1 [file sports-14-00429-s001.zip › Supplementary Material S1.pdf]

Polostrukturovaný rozhovor
Psychologická příprava vrcholových tenistů

Děkuji, že jste si našel/našla čas na tento rozhovor. Zapnutí nahrávky. Informovaný souhlas. Cílem rozhovoru je porozumět tomu, jak vrcholoví tenisté prožívají psychologickou stránku své sportovní kariéry, jaké mají zkušenosti s psychologickou přípravou a jaký význam jí přikládají. Neexistují správné ani špatné odpovědi. Zajímá mě především vaše zkušenost a pohled na věc.

1. Sportovní dráha a kontext

Mohl/a byste mi krátce popsat svou tenisovou kariéru?
Jak aktuálně vypadá vaše sportovní příprava?
Co je podle vás důležité pro úspěch ve vrcholovém tenise?

2. Význam psychologické přípravy

Jaké zkušenosti máte s psychologickou přípravou?
Jakou roli podle vás hraje psychika ve vrcholovém tenise?

3. Zkušenosti s podporou okolí

Jak se k psychologické přípravě staví lidé ve vašem okolí?
Jakou roli v tom hrají trenéři, rodina nebo další členové týmu?

4. Zkušenost před zápasem

Mohl/a byste popsat, co obvykle prožíváte před důležitým zápasem?
Jak vypadá váš den před zápasem?

5. Prožívání během zápasu

Jaké bývá vaše prožívání během zápasu?
Co se obvykle děje ve chvílích, kdy se zápas vyvíjí podle vašich představ?
A co se děje, když se zápas nevyvíjí podle vašich představ?
Doplňující otázky:

- Můžete uvést konkrétní příklad?
- Co jste si v takové chvíli říkal/a?
- Co vám pomohlo situaci zvládnout?
- Jak se vaše prožívání během kariéry změnilo?

6. Náročné momenty a zvládání obtížných situací

Vzpomínáte si na nějaký mimořádně náročný okamžik během zápasu nebo kariéry?

Jak jste tuto situaci prožíval/a? Co vám tehdy pomohlo? Co pro vás tato zkušenost znamenala?

7. Nejsilnější zkušenosti

Vybavíte si nějakou situaci, kdy jste měl/a pocit, že psychická stránka výkonu sehrála zásadní roli?

Můžete mi o této zkušenosti vyprávět?

8. Po zápase

Co obvykle prožíváte po zápase?

Jaké jsou pro vás rozdíly mezi prožitkem po vítězství a po porážce?

Co bývá nejtěžší? Co bývá nejpříjemnější?

9. Doporučení mladým tenistům

Co byste doporučil/a mladým hráčům, kteří se chtějí ve vrcholovém tenise prosadit?

10. Závěr

Je něco důležitého, na co jsem se nezeptala a co by podle vás mělo zaznít?

Je něco, co byste chtěl/a doplnit nebo upřesnit?

Poděkování, rozloučení.
