# Supplementary material for: Mental Preparation in Professional Tennis: A Qualitative Study of Czech Players’ Experiences
Source: Sports (Basel). 2026 Oct 3;14(10):429. doi: 10.3390/sports14100429 (PMC13709374; doi:10.3390/sports14100429)
Supplement: Supplementary file 1 [file sports-14-00429-s001.zip › Supplementary Material S2.pdf]

Semi-Structured Interview

Psychological Preparation of Elite Tennis Players

Thank you for taking the time to participate in this interview. Recording started. Informed consent obtained.
The aim of this interview is to gain an understanding of how elite tennis players experience the psychological aspects of their sporting careers, what experiences they have with psychological preparation, and what meaning they attribute to it. There are no right or wrong answers. I am primarily interested in your personal experiences and perspectives.

1. Tennis Career and Context

- Could you briefly describe your tennis career?
- What does your current training and preparation look like?
- In your opinion, what is most important for success in elite tennis?

2. Meaning of Psychological Preparation

- What experiences have you had with psychological preparation?
- What role do you think psychology plays in elite tennis?

3. Experiences of Support from Others

- How do the people around you perceive psychological preparation?
- What role do coaches, family members, and other members of your support team play in this area?

4. Experiences Before a Match

- Could you describe what you typically experience before an important match?
- What does a typical day before a match look like for you?

5. Experiences During a Match

- What is your experience like during a match?
- What usually happens when a match is going according to your expectations?
- What happens when a match is not going according to your expectations?
- Follow-up questions: Could you give a specific example? What were you thinking in that moment? What helped you manage the situation? How has your experience changed throughout your career?

6. Challenging Moments and Coping with Difficult Situations

- Do you recall a particularly challenging moment during a match or at some point in your career?
- How did you experience that situation? What helped you at the time? What did that experience mean to you?

7. Most Significant Experiences

- Can you think of a situation in which you felt that the psychological side of performance played a crucial role?
- Could you tell me more about that experience?

8. After the Match

- What do you typically experience after a match?
- What differences do you experience between winning and losing?
- What tends to be the most difficult part? What tends to be the most rewarding or enjoyable part?

9. Advice for Young Tennis Players

- What advice would you give to young players who want to succeed in elite tennis?

10. Closing

- Is there anything important that I have not asked about that you think should be discussed?
- Is there anything you would like to add or clarify?

Thank you for sharing your experiences. I appreciate your time and participation. Goodbye.
